# A 3‐Month Follow‐Up Pilot Study on Accelerated Intermittent Theta Burst Stimulation for Bipolar Depression

**DOI:** 10.1111/bdi.70148

**Published:** 2026-07-02

**Authors:** Daan Neuteboom, Urmi Pahladsingh, Martijn C. Steinbach, Marjan C. Ploegaert, Jasper B. Zantvoord, Anja Lok, Lieuwe de Haan, Karel W. F. Scheepstra

**Affiliations:** ^1^ Amsterdam UMC University of Amsterdam, Adult Psychiatry Amsterdam the Netherlands; ^2^ Amsterdam Neuroscience, Mood, Anxiety, Psychosis, Stress & Sleep Amsterdam the Netherlands; ^3^ Parnassia Group the Hague the Netherlands; ^4^ Center for Urban Mental Health University of Amsterdam Amsterdam the Netherlands; ^5^ Neuroimmunology Research Group Netherlands Institute for Neuroscience Amsterdam the Netherlands

**Keywords:** accelerated intermittent theta burst stimulation (aiTBS), bipolar disorder (BD), non‐invasive brain stimulation (NIBS), repetitive transcranial magnetic stimulation (rTMS), treatment resistant bipolar depression (TRBD)

## Abstract

**Background:**

Approximately 25% of patients with bipolar disorder are reported to have treatment‐resistant bipolar depression (TRBD). Accelerated intermittent Theta Burst Stimulation (aiTBS) is an innovative form of repetitive transcranial magnetic stimulation (rTMS), delivering bursts of stimulation at theta wave frequencies, which are believed to enhance synaptic plasticity. This pilot study aimed to explore the safety, tolerability, and preliminary efficacy of aiTBS in individuals with treatment‐resistant bipolar depression (TRBD).

**Methods:**

This open‐label pilot study (registered at Overview of Medical Research in the Netherlands (OMON), NL‐OMON53634), conducted between July 2023 and September 2024, included patients aged 43–64 years who were diagnosed with bipolar I or II and were experiencing a current moderate‐to‐severe depressive episode. Patients were required to have treatment‐refractory symptoms according to the Hidalgo‐Mazzei criteria. All patients were receiving antidepressant medication. Patients received eight daily sessions of intermittent Theta Burst Stimulation (iTBS) over five consecutive days, with 50‐min intervals between sessions. Stimulation targeted the left dorsolateral prefrontal cortex (DLPFC) using the BeamF3 targeting method. Outcomes included safety, tolerability, and efficacy and were assessed at day 3, day 5, week 2, week 4 and week 12 post‐treatment. Safety was assessed by documentation of serious adverse events (SAEs) and adverse events (AEs); tolerability was evaluated based on reported side effects. Efficacy was measured by the mean reduction in depression symptoms using the 17‐item Hamilton Depression Rating Scale (HDRS‐17).

**Results:**

Eight patients were recruited, all of whom completed the treatment course. aiTBS was well tolerated and no SAEs occurred during the treatment week or during follow‐up. The most frequently reported AEs were discomfort at the stimulation site and fatigue (87.5%). Although one patient experienced hypomanic symptoms (YMRS = 13) at the 3‐month follow‐up visit, this was considered unrelated to the study treatment due to the timing of onset, and because several significant psychosocial stressors were identified in the patient's life during that period. Mean HDRS scores decreased from 22.9 (SD = 4.4) at baseline to 16.6 (SD = 4.2) on day 3 of treatment (difference = 6.3 (95% CI, 1.6–10.9); 27.3% improvement, *p* = 0.01), 12.8 (SD = 4.1) on day 5 (difference = 10.1 (95% CI, 2.2–18.1); 44.3%, *p* = 0.02), 11.1 (SD = 3.8) at week 2 (difference = 11.8 (95% CI, 4.5–19.0); 51.4%, *p* = 0.004) and 13.5 (SD = 3.9) at week 4 (difference = 9.4 (95% CI, 1.0–17.7); 41.0% improvement, *p* = 0.03). At month 3, the mean HDRS score was 13.7 (SD = 6.9, difference compared to baseline = 9.1 (95% CI, −3.0 to 21.3); 40.0% improvement, *p* = 0.2).

**Conclusion:**

This pilot study extends prior evidence suggesting the antidepressant effects, safety, and tolerability of aiTBS in patients with TRBD; however, its durability may be limited. We recommend that future RCTs investigate relapse prevention following acute aiTBS and determine the optimal strategy for maintaining antidepressant effects.

## 1 Introduction

In bipolar disorder (BD), depressive episodes are more pervasive and longer‐lasting than (hypo)manic episodes [1, 2]. Bipolar depression is associated with significant psychosocial impairment, elevated suicide risk, and high morbidity and mortality rates [1, 3]. Approximately 25% of patients with bipolar disorder are reported to have treatment‐resistant bipolar depression (TRBD), failing to respond to various mood‐stabilizing treatments [4, 5]. Additionally, the chronic side effects of current pharmacological treatments, including cardiovascular, metabolic, and renal adverse effects, further complicate the management of BD [6, 7]. This emphasizes the urgent need for novel effective treatments with a more favorable side‐effect profile for patients with bipolar depression.

A promising intervention with a comparatively favorable side‐effect profile is accelerated intermittent theta burst stimulation (aiTBS), a modality of non‐invasive brain stimulation (NIBS). Accelerated intermittent theta burst stimulation (aiTBS) is an advanced form of repetitive transcranial magnetic stimulation (rTMS), involving the delivery of theta‐frequency bursts that are thought to enhance synaptic plasticity [8].

A large systematic review and network meta‐analysis by Kishi et al. (2024) compared the safety profile of various TBS protocols with sham treatment [9]. Overall, TBS was well tolerated, and the most common side effects were mild and transient. No significant differences were found in the overall rate of adverse events. Furthermore, a systematic review by Neuteboom et al. (2023) reported emerging evidence that accelerated iTBS is a safe and tolerable form of NIBS with a rapid antidepressant effect in patients with major depressive disorder (MDD) [10].

Additionally, a systematic review and meta‐analysis by Cai et al. (2023) evaluated the clinical efficacy and safety of aiTBS in patients with major depressive disorder or bipolar depression [11]. The analysis included five double‐blind randomized controlled trials comprising 239 patients. The findings revealed that active aiTBS outperformed sham stimulation in terms of response rates, suggesting its potential as an effective treatment modality for TRBD.

Further supporting this evidence, a recent randomized controlled trial by Sheline et al. (2024) found that connectivity‐guided aiTBS was more effective than sham stimulation in 24 patients with TRBD [12]. Patients in the active group showed a significant reduction in Montgomery–Åsberg Depression Rating Scale (MADRS) scores, from 30.4 to 10.5, compared with the sham group, whose scores decreased from 28.0 to 25.3. Adverse events were generally mild, and no serious adverse events were reported.

These results are consistent with the findings of Appelbaum et al. (2025) [13]. In this randomized, double‐blind, sham‐controlled trial of 13 patients with TRBD, active aiTBS produced a substantial within‐group reduction in depressive symptoms (56% reduction in MADRS scores) that was not observed in the sham group. Although between‐group differences did not reach statistical significance, effect sizes were large, and no cases of hypomania or mania occurred, indicating good tolerability. These findings support previous evidence that personalized connectivity‐guided aiTBS may yield rapid and sustained antidepressant effects in bipolar depression.

While the preliminary results are promising, further large‐scale studies are needed to confirm these findings and establish long‐term safety and efficacy profiles. Furthermore, the efficacy and safety of Beam F3 scalp‐based targeting remain unclear, as existing evidence has largely focused on connectivity‐guided aiTBS.

Therefore, we present an open‐label pilot study with a 3‐month follow‐up to assess the safety, tolerability, and preliminary efficacy of accelerated intermittent theta burst stimulation in eight patients with treatment‐resistant bipolar depression, using the Beam F3 method for target localization.

## 2 Methods

### 2.1 Study Design

This open‐label pilot study was approved by the Amsterdam UMC Medical Ethics Review Committee (METC) and registered under number NL83361.018.22. The study is also registered in the Overview of Medical Research in the Netherlands (OMON) under number NL‐OMON53634.

### 2.2 Patients

Patients were recruited between July 2023 and September 2024. Patients were eligible for treatment if they met the following criteria: a DSM‐5 diagnosis of bipolar I or II disorder with a current treatment‐resistant depressive episode (TRBD), defined according to the criteria described by Hidalgo‐Mazzei (i.e., lack of remission for 8 consecutive weeks following two different medication trials at adequate therapeutic doses, including at least two recommended monotherapy treatments or one monotherapy treatment combined with another treatment) [14]; a Hamilton Depression Rating Scale (HDRS‐17) score > 16 at baseline; a stable medication dosage for at least 4 weeks prior to aiTBS initiation; a stable dosage of anti‐manic medication for patients with bipolar I disorder; and no (hypo)manic episode during the 3 months preceding treatment. For exclusion criteria, see the Supporting Informations.

### 2.3 Protocol

Patients received stimulation over the left dorsolateral prefrontal cortex (DLPFC) eight times daily for five consecutive days. The DLPFC was targeted using the Beam F3 method, and pulses were delivered at 100% of the resting motor threshold (rMT). Each stimulation session lasted 10 min and was separated by a 50‐min intersession interval (ISI). Each session consisted of 1800 pulses, resulting in a total of 72,000 pulses over the course of treatment. Patients were admitted to the inpatient psychiatric clinic of Amsterdam UMC for the duration of the intervention.

### 2.4 Primary Outcomes

The primary outcomes were safety, tolerability, and efficacy. Safety was assessed by documenting (serious) adverse events, tolerability by reported side effects, and efficacy by the mean change in HDRS‐17 scores.

Secondary outcomes included response and remission rates (defined as a ≥ 50% reduction in HDRS‐17 score and an HDRS‐17 score ≤ 7, respectively), the Inventory of Depressive Symptomatology–Self‐Report (IDS‐SR), and the Young Mania Rating Scale (YMRS).

All assessments were performed at baseline, day 3 and day 5 of treatment, and at week 2, week 4, and month 3 following treatment initiation.

### 2.5 Statistical Analysis

Changes in HDRS‐17 and YMRS scores were analyzed using repeated‐measures one‐way ANOVA. There were no missing data for these measures. Tukey's post hoc test was used to correct for multiple comparisons. Because IDS‐SR data contained missing values, a mixed‐effects model using the restricted maximum likelihood method was applied. Tukey's post hoc test was again used to correct for multiple comparisons. The data were assumed to follow a Gaussian distribution, and the Geisser–Greenhouse correction was applied to correct for Type I error. Findings with a *p*‐value < 0.05 were considered statistically significant. All analyses were performed using GraphPad Prism version 9.5.1 (San Diego, CA, United States).

## 3 Results

In total, eight patients (7 female, 1 male) with a mean age of 53.4 years (range 43–64; SD = 7.5) were treated with aiTBS. Six patients were diagnosed with bipolar II disorder and two with bipolar I disorder. The mean duration of the current depressive episode was 11.9 months (range 2–24; SD = 7.2). The mean number of previous medication trials was 6.0 (range 2–10; SD = 3.4). For all demographic and clinical characteristics, see Table S1.

### 3.1 Safety and Tolerability

No SAEs occurred during the treatment week. The most commonly reported adverse events were discomfort at the stimulation site and fatigue (87.5%), followed by headache (62.5%). Overall, adverse events were limited to the treatment week. No treatment‐emergent (hypo)mania was observed during the intervention week or follow‐up. However, one patient experienced hypomanic symptoms (YMRS = 13) at the 3‐month follow‐up visit.

Due to discomfort at the stimulation site, one patient received stimulation at 90% rMT. Another patient shortened several sessions because of discomfort at the stimulation site, dizziness, and general ambivalence toward treatment. In total, 7 of 40 scheduled sessions, distributed across days 3, 4, and 5 of the intervention week, were missed. For all side effects observed during the treatment week, see Table S2.

### 3.2 Reduction in Depressive Symptoms

**FIGURE 1:**
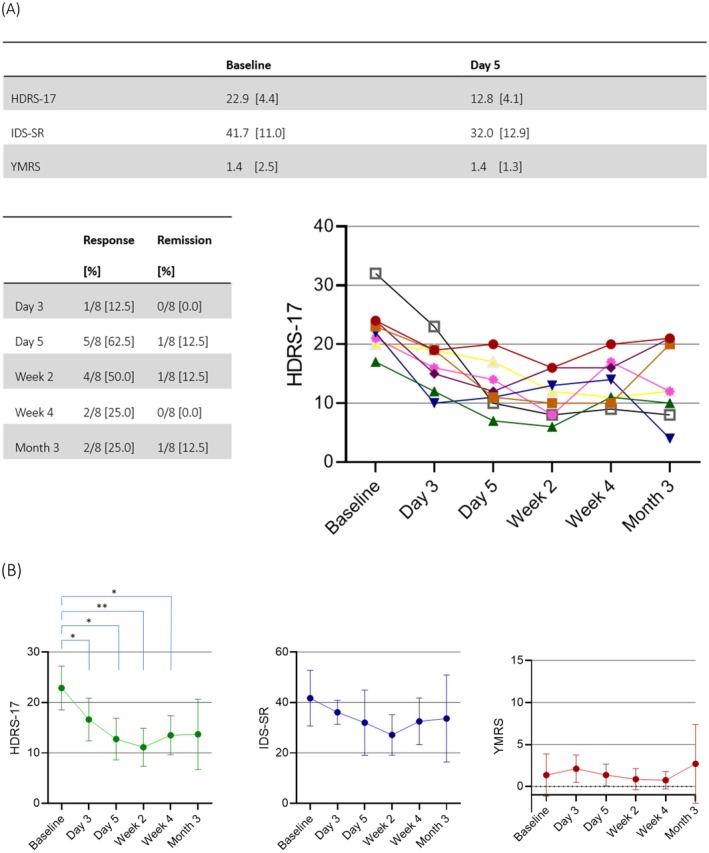
Overview of baseline characteristics, outcome measures, response and remission rates, and individual HDRS‐17 score trajectories throughout the study. (A) Baseline characteristics, outcome measures at post‐treatment and follow‐up assessments, response and remission rates, and individual HDRS‐17 score trajectories. (B) Mean HDRS‐17, IDS‐SR, and YMRS scores during aiTBS treatment and throughout the follow‐up period. HDRS‐17, Hamilton Depression Rating Scale–17 items; IDS‐SR, Inventory of Depressive Symptomatology–Self‐Report; YMRS, Young Mania Rating Scale. *Statistically significant compared with baseline. **p < 0.005.

See Figure 1 for outcome measures, response rates, and remission rates throughout treatment and follow‐up. Mean HDRS scores decreased significantly from 22.9 (SD = 4.4) at baseline to 16.6 (SD = 4.2) on day 3 of treatment (difference = 6.3 [95% CI, 1.6–10.9]; 27.3% improvement; SE = 1.2; *p* = 0.01), to 12.8 (SD = 4.1) on day 5 (difference = 10.1 [95% CI, 2.2–18.1]; 44.3% improvement; *p* = 0.02), to 11.1 (SD = 3.8) at week 2 of follow‐up (difference = 11.8 [95% CI, 4.5–19.0]; 51.4% improvement; *p* = 0.004), and to 13.5 (SD = 3.9) at week 4 of follow‐up (difference = 9.4 [95% CI, 1.0–17.7]; 41.0% improvement; *p* = 0.03). At month 3 of follow‐up, the mean HDRS score was 13.7 (SD = 6.9); however, this did not differ significantly from baseline (difference = 9.1 [95% CI, −3.0 to 21.3]; 40.0% improvement; *p* = 0.20).

At day 5, week 2 of follow‐up, week 4 of follow‐up, and month 3 of follow‐up, response rates were 62.5%, 50.0%, 25.0%, and 25.0%, respectively. Remission rates at these time points were 12.5%, 12.5%, 0.0%, and 12.5%, respectively. Tables S3–S5 provide all outcome measures, including IDS‐SR and YMRS scores.

## 4 Discussion

We investigated the safety, tolerability, and preliminary efficacy of administering eight daily aiTBS sessions over five consecutive days in patients with treatment‐resistant bipolar depression. We found that this protocol was safe and well tolerated. No serious adverse events occurred, and the most common side effects were discomfort at the stimulation site, fatigue, and headache. Importantly, no treatment‐emergent (hypo)mania occurred during the treatment week. One patient developed hypomanic symptoms at the 3‐month follow‐up. In addition, we observed significant reductions in depressive symptoms immediately after treatment, which were maintained up to 1 month post‐treatment.

Consistent with previous studies, aiTBS was generally well tolerated, and no SAEs were observed during the intervention week [9, 10, 11, 12, 13, 15]. One patient with bipolar II disorder exhibited hypomanic symptoms (YMRS = 13) at the 3‐month follow‐up assessment. This episode was considered unlikely to be directly attributable to the intervention, as several significant psychosocial stressors were present during this period. These contextual factors may have contributed to the elevated YMRS score, together with a concurrent reduction in HDRS‐17 severity. Alternatively, the episode may reflect the natural course of bipolar disorder. Importantly, in line with previous findings, no treatment‐emergent hypomanic symptoms were observed during the intervention week or within the first 4 weeks following treatment [9, 10, 11, 12, 13, 15].

Regarding clinical outcomes, the present study demonstrated a 44.3% reduction in HDRS‐17 scores immediately post‐treatment, a 51.4% reduction at 2 weeks, and a 41.0% reduction at 4 weeks. Appelbaum et al. (2025) reported reductions in MADRS scores of 56.8% and 55.2% at one and 4 weeks post‐treatment, respectively [13], whereas Sheline et al. (2024) reported HDRS‐17 reductions of 64.0% and 69.0% at day 1 and week 4 post‐treatment, respectively [12]. Differences in treatment response across studies may be partially explained by variations in stimulation parameters, including the number of daily sessions and methods of target localization.

In the studies by Appelbaum et al. and Sheline et al., patients received personalized image‐guided aiTBS for 5 days at 90% of the resting motor threshold (rMT), with 10 daily sessions totaling 90,000 pulses. Both the number of daily sessions and the cumulative pulse count exceeded those used in the present study, which delivered eight sessions per day over 5 days (72,000 pulses in total). This comparatively lower stimulation dose may be suboptimal for individuals with treatment‐resistant bipolar depression, who may require a higher cumulative dose to achieve robust and sustained antidepressant effects [16, 17].

Target localization may further contribute to differences in treatment outcomes. Whereas the current study used the Beam F3 method to target the left dorsolateral prefrontal cortex (DLPFC), previous studies employed neuroimaging‐guided targeting, which may enable more precise stimulation of functionally relevant cortical regions and thereby enhance treatment efficacy [18]. To date, no randomized controlled trials have directly compared these targeting strategies.

Consistent with earlier aiTBS research, several initial responders in our sample experienced a loss of therapeutic benefit by the 3‐month follow‐up assessment [9, 10, 11, 12, 13, 15]. These findings suggest that although aiTBS may induce rapid antidepressant effects, the durability of these effects may be limited. Future studies should evaluate the utility of maintenance aiTBS protocols or tapering schedules to sustain clinical improvements over extended periods.

Despite these encouraging findings, several limitations warrant consideration. The small sample size, open‐label design, absence of a control group, and missing data (e.g., IDS‐SR scores) restrict the interpretability and generalizability of the results. Additionally, the sample included only two patients with bipolar I disorder, and potential confounders—such as inpatient admission and concurrent psychosocial stressors—may also have influenced outcomes.

Nevertheless, the observed antidepressant effects and overall tolerability of aiTBS contribute to the growing body of evidence supporting its potential utility in affective disorders. These preliminary findings underscore the need for larger randomized controlled trials with appropriate sham conditions to systematically evaluate the short‐ and long‐term safety, tolerability, and efficacy of aiTBS in patients with bipolar depression. Future research should also investigate the neurobiological mechanisms underlying treatment response, with particular attention to optimizing stimulation parameters and identifying biomarkers associated with durable clinical improvement.

## Conflicts of Interest

The authors declare no conflicts of interest.

## Supporting information


**Table S1:** Patient characteristics and baseline psychometrics.
**Table S2:** Overview of side effects.
**Table S3:** aiTBS parameters and clinical assessments.
**Table S4:** Mean Difference HDRS‐17 between baseline and timepoints.
**Table S5:** Mean Difference IDS‐SR between baseline and timepoints.

## Data Availability

The data that supports the findings of this study are available in the Supporting Information of this article.

## References

[1] I. Grande , M. Berk , B. Birmaher , and E. Vieta , “Bipolar Disorder,” Lancet (London, England) 387, no. 10027 (2016): 1561–1572, 10.1016/S0140-6736(15)00241-X. 26388529

[2] L. Tondo , G. H. Vázquez , and R. J. Baldessarini , “Depression and Mania in Bipolar Disorder,” Current Neuropharmacology 15, no. 3 (2017): 353–358, 10.2174/1570159X14666160606210811. 28503106 PMC5405618

[3] R. J. Baldessarini , P. Salvatore , H. M. Khalsa , et al., “Morbidity in 303 First‐Episode Bipolar I Disorder Patients,” Bipolar Disorders 12, no. 3 (2010): 264–270, 10.1111/j.1399-5618.2010.00812.x. 20565433

[4] J. Mendlewicz , I. Massat , S. Linotte , et al., “Identification of Clinical Factors Associated With Resistance to Antidepressants in Bipolar Depression: Results From an European Multicentre Study,” International Clinical Psychopharmacology 25, no. 5 (2010): 297–301, 10.1097/YIC.0b013e32833c4ceb. 20571412

[5] M. Fornaro , A. F. Carvalho , A. Fusco , et al., “The Concept and Management of Acute Episodes of Treatment‐Resistant Bipolar Disorder: A Systematic Review and Exploratory Meta‐Analysis of Randomized Controlled Trials,” Journal of Affective Disorders 276 (2020): 970–983, 10.1016/j.jad.2020.07.109. 32750614

[6] J. G. Fiedorowicz , N. M. Palagummi , V. L. Forman‐Hoffman , D. D. Miller , and W. G. Haynes , “Elevated Prevalence of Obesity, Metabolic Syndrome, and Cardiovascular Risk Factors in Bipolar Disorder,” Annals of Clinical Psychiatry: Official Journal of the American Academy of Clinical Psychiatrists 20, no. 3 (2008): 131–137, 10.1080/10401230802177722. 18633739 PMC2776768

[7] D. J. Smith , D. Martin , G. McLean , J. Langan , B. Guthrie , and S. W. Mercer , “Multimorbidity in Bipolar Disorder and Undertreatment of Cardiovascular Disease: A Cross Sectional Study,” BMC Medicine 11 (2013): 263, 10.1186/1741-7015-11-263. 24359325 PMC3880052

[8] Y. Z. Huang , M. J. Edwards , E. Rounis , K. P. Bhatia , and J. C. Rothwell , “Theta Burst Stimulation of the Human Motor Cortex,” Neuron 45, no. 2 (2005): 201–206, 10.1016/j.neuron.2004.12.033. 15664172

[9] T. Kishi , T. Ikuta , K. Sakuma , et al., “Theta Burst Stimulation for Depression: A Systematic Review and Network and Pairwise Meta‐Analysis,” Molecular Psychiatry 29, no. 12 (2024): 3893–3899, 10.1038/s41380-024-02630-5. 38844532 PMC11609094

[10] D. Neuteboom , J. B. Zantvoord , R. Goya‐Maldonado , et al., “Accelerated Intermittent Theta Burst Stimulation in Major Depressive Disorder: A Systematic Review,” Psychiatry Research 327 (2023): 115429, 10.1016/j.psychres.2023.115429. 37625365

[11] D. B. Cai , Z. J. Qin , X. J. Lan , et al., “Accelerated Intermittent Theta Burst Stimulation for Major Depressive Disorder or Bipolar Depression: A Systematic Review and Meta‐Analysis,” Asian Journal of Psychiatry 85 (2023): 103618, 10.1016/j.ajp.2023.103618. 37201381

[12] Y. I. Sheline , W. Makhoul , A. S. Batzdorf , et al., “Accelerated Intermittent Theta‐Burst Stimulation and Treatment‐Refractory Bipolar Depression: A Randomized Clinical Trial,” JAMA Psychiatry 81, no. 9 (2024): 936–941, 10.1001/jamapsychiatry.2024.1787. 38985492 PMC11238064

[13] L. G. Appelbaum , H. Daniels , L. Lochhead , et al., “Accelerated Intermittent Theta‐Burst Stimulation for Treatment‐Resistant Bipolar Depression: A Randomized Clinical Trial,” JAMA Network Open 8, no. 2 (2025): e2459361, 10.1001/jamanetworkopen.2024.59361. 39932714 PMC11815521

[14] D. Hidalgo‐Mazzei , M. Berk , A. Cipriani , et al., “Treatment‐Resistant and Multi‐Therapy‐Resistant Criteria for Bipolar Depression: Consensus Definition,” British Journal of Psychiatry: The Journal of Mental Science 214, no. 1 (2019): 27–35, 10.1192/bjp.2018.257. 30520709 PMC7613090

[15] K. Li , A. Bichlmeier , C. DuPont , et al., “Fast Depressive Symptoms Improvement in Bipolar I Disorder After Stanford Accelerated Intelligent Neuromodulation Therapy (SAINT): A Two‐Site Feasibility and Safety Open‐Label Trial,” Journal of Affective Disorders 365 (2024): 359–363, 10.1016/j.jad.2024.08.087. 39154984 PMC11495146

[16] P. B. Fitzgerald , K. E. Hoy , J. Reynolds , et al., “A Pragmatic Randomized Controlled Trial Exploring the Relationship Between Pulse Number and Response to Repetitive Transcranial Magnetic Stimulation Treatment in Depression,” Brain Stimulation 13, no. 1 (2020): 145–152, 10.1016/j.brs.2019.09.001. 31521543

[17] L. Schulze , K. Feffer , C. Lozano , et al., “Number of Pulses or Number of Sessions? An Open‐Label Study of Trajectories of Improvement for Once‐Vs. Twice‐Daily Dorsomedial Prefrontal rTMS in Major Depression,” Brain Stimulation 11, no. 2 (2018): 327–336, 10.1016/j.brs.2017.11.002. 29153439

[18] R. F. H. Cash , A. Weigand , A. Zalesky , et al., “Using Brain Imaging to Improve Spatial Targeting of Transcranial Magnetic Stimulation for Depression,” Biological Psychiatry 90, no. 10 (2021): 689–700, 10.1016/j.biopsych.2020.05.033. 32800379

