# Supplementary material for: A 3‐Month Follow‐Up Pilot Study on Accelerated Intermittent Theta Burst Stimulation for Bipolar Depression
Source: Bipolar Disord. 2026 Jul 2;28(5):e70148. doi: 10.1111/bdi.70148 (PMC13329208; doi:10.1111/bdi.70148)
Supplement: Supplementary file 1 — Table S1: Patient characteristics and baseline psychometrics. Table S2: Overview of side effects. Table S3: aiTBS parameters and clinical assessments. Table S4: Mean Difference HDRS‐17 between baseline and timepoints. Table S5: Mean Difference IDS‐SR between baseline and timepoints. [file BDI-28-0-s001.docx]

**Supplementary materials**

Exclusion criteria:

Participants were excluded if they met the following criteria:

- In BPD, (hypo) manic episode within 3 months prior start of the trial;
- In BPD, Young Mania Rating Scale (YMRS) score of >12 prior start of the trial;
- Patients with bipolar II disorder who use anti-depressant medication without anti-manic medication or patients with bipolar I disorder, not using anti-manic medication;
- Inability to understand or comply with study requirements as judged by the investigators, assessed at the baseline interview;
- Acute suicidality;
- (History of) neurological disease
- (History of) epilepsy or epilepsy in a 1^st^ degree relative;
- Pregnancy;
- Abnormal hearing or (uncorrected) vision;
- Disorder in substance abuse (opiate, ketamine, LSD, (meth)amphetamine, cocaine, solvents, cannabis, benzodiazepines or barbiturate) or alcohol abuse;
- Known serious somatic health problem;
- Used recreational drugs over a period of 72 hours prior each session;
- Used alcohol within the last 24 hours prior each session;
- Specific TMS contraindications (see standard screening form, appendix 1): ICD, pacemaker, history of epileptic seizures of epilepsy in a 1^st^ degree relative, intracranial metal implants(e.g. cochlear implant or deep brain stimulator).

| **Supplementary Table 1.** Patient characteristics and baseline psychometrics | | | | | | | | | | |
| --- | --- | --- | --- | --- | --- | --- | --- | --- | --- | --- |
| **Patient ID** | | | | | | | | | | |
|  | **1** | **2** | **3** | **4** | | **5** | **6** | **7** | **8** |  |
| **General characteristics** | | | | | | | | | | |
| Gender | F | F | F | F | M | | F | F | F | 7F/1M |
| Bipolar type | I | II | II | II | II | | II | II | II | 1 type I/  7 type II |
| Age at entry (years)** | - | - | - | - | - | | - | - | - | 53.4 (±7.5) |
| **Psychiatric history** | | | | | | | | | | |
| Age at onset (years) | 20 | 43 | 40 | 34 | 24 | | 22 | 46 | 53 | 35.3  (± 12.2) |
| Length of diagnosis (years) | 45 | 1 | 5 | 28 | 28 | | 36 | 8 | 2 | 19.3  (± 17.1) |
| Length of current depressive episode (months) | 13 | 13 | 12 | 2 | 10 | | 24 | 3 | 18 | 11.9  (± 7.2) |
| **Episode specifiers** | | | | | | | | | | |
| Psychotic features | - | - | - | - | Yes | | - | **-** | **-** | 1/8 |
| **Previous treatment** | | | | | | | | | | |
| ECT | Yes | No | No | No | Yes | | No | No | N0 | 2 Yes /  6 No |
| Number of medication trials* | 9 | 8 | 2 | 4 | 10 | | 3 | 5 | 8 | 6.0 (±3.4) |
| **Current medication** | | | | | | | | | | |
| SSRI/SNRI/NDRI^S^ | - | 1 | 1 | - | 1 | | - | 1 | - |  |
| TCA^T^ | - | - | - | - | - | | - | 1 | 1 |  |
| Antipsychotics^A^ | 1 | 1 | - | 1 | - | | 1 | 1 | 1 |  |
| Mood stabilizers^M^ | - | - | 1 | 1 | 1 | | 2 | 1 | 1 |  |
| Benzodiazepines ^B^ | - | 1 | 1 | 2 | 1 | | - | 1 | 2 |  |

 BD = bipolar disorder, HDRS-17 = Hamilton Depression Rating Scale – 17 items, IDS-SR = Inventory of Depressive Symptomology – Self-Report

 *Medication trials encompass all past and present antipsychotic, antidepressive and mood stabilizing medication, which were prescribed to treat the bipolar disorder.

** For privacy reasons only the mean age is reported.

^S^Selective Serotonin Reuptake Inhibitors/Seretonin-Norepinephrine Reuptake Inhibitors/ Norepinephrine-Dopamine Reuptake Inhibitors; including escitalopram, venlafaxine and bupropion

^T^Tricyclic antidepressants; including amitriptyline, nortriptyline and clomipramine.

^A^Antipsychotics; including quetiapine and olanzapine.

^M^Mood stabilizers; including lithium and lamotrigine.

^B^Benzodiazepines; including lorazepam, alprazolam, lormetazepam and zolpidem.

| **Supplementary Table 2.** Overview of side effects | | | | | | | | | |
| --- | --- | --- | --- | --- | --- | --- | --- | --- | --- |
| **Patient ID** | **1** | **2** | **3** | **4** | **5** | **6** | **7** | **8** |  |
| Discomfort at stimulation site | Yes* | No | Yes* | Yes* | Yes* | Yes* | Yes* | Yes* | 7/8 |
| Fatigue | Yes* | No | Yes* | Yes* | Yes* | Yes* | Yes* | Yes* | 7/8 |
| Headache | Yes* | No | No | No | Yes* | Yes* | Yes^a^ | Yes* | 5/8 |
| Concentration issues | No | No | No | No | No | Yes* | Yes* | No | 2/8 |
| Dizziness | No | No | No | No | Yes* | No | No | No | 1/8 |
| Nausea | No | No | No | No | No | Yes* | No | Yes* | 1/8 |
| Tingling in extremities | No | No | No | No | No | Yes* | No | No | 1/8 |
| Other** | No | No | No | No | No | No | Yes* | No | 1/8 |

* Side effects were limited to treatment days.

^a^Patient 7 experienced headache throughout follow-up, however the patient suffered from headaches prior to the treatment.

** Difficulty coming up with words while speaking

| **Supplementary Table 3.** aiTBS parameters and clinical assessments    **Patient ID** | | | | | | | | | |
| --- | --- | --- | --- | --- | --- | --- | --- | --- | --- |
|  | **1** | **2** | **3** | **4** | **5** | **6** | **7** | **8** |  |
| **aiTBS parameters** | | | | | | | | | |
| Number  of sessions | 40 | 40 | 40 | 40 | 33 | 40 | 40 | 40 | 38.8 (±2.9) |
| Stimulation intensity | 42.4 (±1.7) | 44.8 (±0.4) | 50.0 (±2.0) | 47.4 (±2.1) | 43.6  (±2.5) | 50  (±0) | 38.6  (±2.1) | 45.4  (±0.9) |  |
| **Clinical assessments** | | | | | | | | | |
| **Baseline** | | | | | | | | | |
| HDRS-17 | 24 | 23 | 17 | 22 | 24 | 21 | 32 | 20 | 22.9 (±4.4) |
| IDS-SR | 48 | Missing Data | 28 | 45 | 24 | 51 | 50 | 46 | 41.7 (±11.0) |
| YMRS | 0 | 1 | 0 | 0 | 3 | 0 | 0 | 7 | 1.4 (±2.5) |
| **Day 3** | | | | | | | | | |
| HDRS-17 | 19 | 19 | 12 | 10 | 15 | 16 | 23 | 19 | 16.6 (±4.2) |
| IDS-SR | 42 | 30 | 33 | 37 | 31 | 38 | 35 | 43 | 36.1 (±4.8) |
| YMRS | 0 | 1 | 5 | 2 | 2 | 1 | 1 | 4 | 2.1 (±1.6) |
| **Day 5** | | | | | | | | | |
| HDRS-17 | 20 | 11 | 7 | 11 | 12 | 14 | 10 | 17 | 12.8 (±4.1) |
| IDS-SR | 40 | 34 | 23 | 43 | 18 | 44 | 11 | 43 | 32.0 (±12.9) |
| YMRS | 0 | 0 | 4 | 1 | 2 | 2 | 1 | 2 | 1.4 (±1.3) |
| **2-week follow up** | | | | | | | | | |
| HDRS-17 | 16 | 10 | 6 | 13 | 16 | 8 | 8 | 12 | 11.1 (±3.8) |
| IDS-SR | 42 | 25 | 30 | Missing Data | Missing Data | 22 | 20 | 24 | 27.2 (±8.0) |
| YMRS | 0 | 0 | 0 | 2 | 3 | 2 | 0 | 0 | 0.9 (±1.2) |
| **4-week follow up** | | | | | | | | | |
| HDRS-17 | 20 | 10 | 11 | 14 | 16 | 17 | 9 | 11 | 13.5 (±3.9) |
| IDS-SR | 40 | 24 | Missing Data | Missing Data | Missing Data | 41 | 25 | 20 | 32.5(±9.3) |
| YMRS | 0 | 0 | 2 | 2 | 2 | 0 | 0 | 0 | 0.8 (±1.0) |
| **3-month follow up** | | | | | | | | | |
| HDRS-17 | 21 | 20 | 10 | 4 | 21 | 12 | 8 | 12 | 13.7(±6.9) |
| IDS-SR | 39 | 49 | 33 | 7 | Missing Data | 39 | 25 | 23 | 32.0 (±14.5) |
| YMRS | 0 | 0 | 1 | 13 | 3 | 0 | 2 | 0 | 2.7 (±4.7) |

BD = bipolar disorder, HDRS-17 = Hamilton Depression Rating Scale – 17 items, IDS-SR = Inventory of Depressive Symptomology – Self-Report, YMRS; Young Mania Rating Scale.

| **Supplementary Table 4.** Mean Difference HDRS-17 between baseline and timepoints | | | |
| --- | --- | --- | --- |
|  | **Mean Difference** | **Mean reduction in percentage [%]** | **P-value, Tukey’s multiple comparisons test** |
| Baseline vs. Day 3 | 6.3 [95% CI, 1.6 – 10.9] | 27.3 | 0.012* |
| Baseline vs. Day 5 | 10.1 [95% CI, 2.2 – 18.1] | 44.3 | 0.015* |
| Baseline vs. Week 2 | 11.8 [95% CI, 4.5 – 19.0] | 51.4 | 0.0039** |
| Baseline vs. week 4 | 9.4 [95% CI, 1.0 – 17.7] | 41.0 | 0.029* |
| Baseline vs. Month 3 | 9.1 [95% CI, -3.0 – 21.3] | 40.0 | 0.15 |

*Statistical significant

| **Supplementary Table 5.** Mean Difference IDS-SR between baseline and timepoints | | | |
| --- | --- | --- | --- |
|  | **Mean Difference** | **Mean reduction in percentage [%]** | **P-value, Tukey’s multiple comparisons test** |
| Baseline vs. Day 3 | 5.6 [95% CI, -6.7 – 17.9] | 13.4 | 0.52 |
| Baseline vs. Day 5 | 9.7 [95% CI, -9.2 – 28.6] | 23.3 | 0.41 |
| Baseline vs. Week 2 | 14.6 [95% CI, -12.7 – 41.7] | 34.9 | 0.29 |
| Baseline vs. week 4 | 11.7 [95% CI, -15.4 – 38.8] | 28.1 | 0.35 |
| Baseline vs. Month 3 | 11.0 [95% CI, -15.3 – 37.3] | 26.3 | 0.54 |
